# Up-regulation of PCSK9 gene expression and diminished level of LDL-receptor in rat liver as a potential cause of post-lipectomy hypercholesterolemia

**DOI:** 10.1007/s11010-018-3484-8

**Published:** 2018-11-27

**Authors:** Agnieszka Dettlaff-Pokora, Elzbieta Sucajtys-Szulc, Tomasz Sledzinski

**Affiliations:** 1 Department of Biochemistry, Faculty of Medicine, Medical University of Gdansk, Debinki 1, 80-211 Gdansk, Poland; 2 Department of Nephrology, Transplantology and Internal Medicine, Faculty of Medicine, Medical University of Gdansk, ul. Debinki 7, 80-211 Gdansk, Poland; 3 Department of Pharmaceutical Biochemistry, Faculty of Pharmacy, Medical University of Gdansk, Debinki 1, 80-211 Gdansk, Poland

**Keywords:** HNF1α, PCSK9, LDL-R, Hypercholesterolemia, Lipectomy

## Abstract

Studies designed to examine effects of fat mass reduction (including lipodystrophy and lipectomy) on human serum total and LDL-cholesterol concentrations are inconsistent. The purpose of this study was to examine effect of partial lipectomy in rats (as an experimental model of fat mass reduction in humans) on (1) circulating total cholesterol, LDL-cholesterol + VLDL-cholesterol and HDL-cholesterol concentrations, and (2) factors which may affect serum cholesterol concentrations such as: (a) liver LDL-receptor level, (b) expression of liver PCSK9 and (c) circulating PCSK9 concentration. Reduction of rat adipose tissue mass resulted in an increase in circulating total and LDL + VLDL—cholesterol concentrations, which was associated with (a) decrease in liver LDL-R level, (b) increase in liver PCSK9 expression, and (c) increase in circulating PCSK9 concentration as compared with sham controls. These changes were accompanied by elevated liver HNF1α (and HNF4α) mRNA levels. Silencing HNF1α in HepG2 cells by siRNA led to decrease in PCSK9 mRNA levels. This suggests that overexpression of HNF1α gene in liver of lipectomized rats can lead to overproduction of PCSK9. In conclusion, up-regulation of PCSK9, due to overexpression of HNF1α gene in liver of lipectomized rats and subsequently increase in circulating PCSK9 concentration lead to decrease in liver LDL-R level. This may contribute, at least in part, to an increase in the concentration of circulating cholesterol in rats with reduced fat mass. These findings provide a possible explanation for the molecular mechanism of hypercholesterolemia observed sometimes after reduction of fat mass in human.

## Introduction

Liposuction is popular esthetic surgery to remove significant amount of subcutaneous adipose tissue within a short period of time, recommended by the American Academy of Cosmetic Surgery [1]. However, the metabolic consequences of liposuction or abdominal lipectomy, especially changes in serum lipids concentrations, are still controversial [2]. The results of previous studies performed in humans have shown: (a) no change [3–7], (b) decrease [8–11], or (c) increase [12] in one or few cardiovascular risk factors including dyslipidemia.

Conflicting results regarding dyslipidemia characterized by: (a) elevated serum total and LDL-cholesterol, (b) elevated serum TAGs and (c) reduction of serum HDL-cholesterol in patients with lipodystrophy were also observed [13–15]. Moreover, individuals with lipodystrophy are at significantly increased risk of atherosclerosis and its consequences like heart disease [16]. Also the results concerning the effect of liposuction on serum cholesterol levels are inconsistent [3–11]. Thus, further studies are needed to clarify the effect of fat loss on lipid metabolism at the molecular level. It seems that, the lipectomy model offers some insight into how fat mass reduction may affect hypercholesterolemia sometimes observed in individuals after weight loss.

Lipectomy performed in obese rat (obesity induced by animal feeding with high fat and high cholesterol diet) had no significant effect on serum concentration of total, LDL- and HDL- cholesterol, but caused higher concentrations of TAGs [17]. Recently we have shown, that partial surgical removal of white adipose tissue in rats is associated with the parallel up-regulation of liver HNF1α (and HNF4α) and genes encoding proteins involved in synthesis, assembly and secretion of TAGs (including ApoB-100 and microsomal triglyceride transfer protein—MTP) as well as serum TAGs concentration [18]. Up-regulation of gene encoding HNF4α was also observed by Ling et al. [17] in lipectomized rats as compared to control animals. Since VLDL and LDL contain ApoB-100 and plasma ApoB-100 concentrations correlates with plasma LDL-cholesterol concentrations [19], one can suppose that increased apoB-100 synthesis and its plasma concentration is also associated with elevated cholesterol concentrations in lipectomized rats.

It is well documented that HNF1α and HNF4α contribute to enhanced expression of genes encoding: (a) ApoB-100 [20, 21] and (b) proprotein convertase subtilisin/kexin 9 (PCSK9) in liver, what is associated with an increase in serum PCSK9 concentrations and consequently with elevated serum cholesterol concentration [22–26]. PCSK9 plays a pivotal role in posttranslational regulation of LDL-R level and subsequently regulation of serum LDL-cholesterol concentration [27]. Several papers reported a positive correlation between concentrations of PCSK9 and LDL-cholesterol in circulation [28–31]. Loss-of-function mutations in human HNF4α cause maturity—onset diabetes of the young type 1 (MODY1) and decrease of plasma cholesterol concentrations [32–35]. Moreover HNF4α is an upstream activator of HNF1α [36], which in turn affects the expression of genes involved in cholesterol and other lipids metabolism [37, 38]. Thus, reciprocal relationship between HNF1α and HNF4α [39, 40] may play important role in regulation of serum cholesterol concentrations.

The aim of this study was to verify if an increase in HNF1α and HNF4α gene expression in the liver is associated with activation of PCSK9 production, which through degradation of LDL-R may lead to increase in serum cholesterol concentrations in lipectomized rats.

## Methods

### Animals and surgeries

The rats were fed ad libitum with standard commercial chow (Laborfed, Poland). Briefly, 12-week-old male Wistar rats were randomly divided into two groups: (1) lipectomized rats (*n* = 10) subjected to resection of epidydymal and retroperitoneal WAT, and (2) controls (*n* = 10) that underwent sham surgery: anesthesia and incision of the skin and muscles without the removal of WAT. After 30 days, the lipectomized rats were anesthetized again with subsequent removal of subcutaneous WAT, and the controls were subjected to another sham surgery. Mean weight of WAT removed from the lipectomized rats was 7.7 ± 0.6 g (3.8 ± 0.3 g, 2.0 ± 0.4 g, and 1.9 ± 0.4 g for inguinal, epidydymal, and retroperitoneal WAT, respectively). The lipectomy was performed as the two-step procedure in order to reduce perioperative mortality and the surgeries were conducted carefully to avoid bleeding. All animals received human care in compliance with the guidelines for the protection of animals used for scientific purposes (Directive, 2010/63 EU, Decision, 2012/707/UE and RD 53/2013). All the procedures involving animals and their care were approved by the Institutional Ethics Committee. The rats were anesthetized and killed by decapitation (between 8:00 a.m. and 10:00 a.m.) after 90 days from the first surgery. Blood samples from the carotid artery were collected to the tubes without anticoagulant, centrifuged at 3000 × *g* for 15 min at 4 °C, and the serum was stored at − 80 °C. At the end of experiments the liver fragments were obtained, immediately frozen in liquid nitrogen and stored at − 80 °C until analysis. Epidydymal, retroperitoneal and inguinal WAT from the controls, as well as the residual WAT from the lipectomized animals, were removed and immediately weighted.

### Serum PCSK9 determination

Commercial ELISA kit was used to estimate PCSK9 in rat serum (CSB-EL017647RA, CUSABIO). Assay was performed according to the manufacturer instruction.

### Serum cholesterol concentrations assays

Serum total cholesterol concentration was determined using a routine method, at the Central Clinical Laboratory, Medical University of Gdansk. Serum HDL- and LDL- + VLDL-cholesterol was measured using HDL and LDL/VLDL Quantitation Kit (Sigma, MAK045). Serum samples were mixed with Precipitation Buffer (2:1), incubated for 10 min at room temperature and centrifuged at 2000 × *g* for 10 min. The precipitate contains LDL/VLDL and the supernatant HDL fractions of cholesterol. Sediment was collected and centrifuged again to remove all remaining trace HDL supernatant. Precipitate was resuspended in Phosphate Buffered Saline (PBS) and the rest of the experiment was performed according to the manufacturer instruction.

### Liver cholesterol assays

Total liver cholesterol, cholesterol esters and free cholesterol were measured using Cholesterol Quantitation Kit (Sigma, MAK043). In brief: 10 mg of liver tissue was extracted with 200 µL of chloroform:isopropanol:IGEPAL CA-630 (7:11:0.1) (Sigma, I8896) in a microhomogenizer. Samples were centrifuged for 10 min at 13,000 × *g* in order to remove insoluble material. Organic phase was transferred to a new tube and dried at 50 °C and further placed under vacuum for 30 min to remove all organic solvents. Dried lipids were resolved in 200 µL of the Cholesterol Assay Buffer, vortexed and sonicated until mixture became homogenous. Cholesterol was measured using fluorometric method (λex = 535/λem = 587 nm). Cholesterol esterase was used to hydrolyze cholesterol esters in order to measure cholesterol esters and total cholesterol in samples tested.

### Cell culture and small interfering RNA (siRNA) transfection

Human hepatocellular carcinoma cell line HepG2 was obtained from ATCC (ATCC; Manassas, VA). Cells were maintained in standard Minimum Essential Eagle’s Medium (MEM; Sigma) with the addition of 2 mM glutamine, 1% non-essential amino acids, 10% fetal bovine serum, penicillin (100 IU per mL), and streptomycin (100 µg per mL). Prior to small interfering RNA (siRNA) transfection, cells were passaged in 6-well plates at 10^5^ cells per well and cultured at 37 °C and grown to approximately 70% confluence. Two different sequences of siRNA targeting HNF1α were used: (a) Hs-TCF1-2, No SI00011620, and (b) Hs-TCF1-5, No SI03095015. AllStars Negative Control, No 1027280 was used as negative control (siRNA NC). All siRNAs were obtained from Qiagen (Crawley, UK). HepG2 cells treated by lipofectamine were used as controls (CON). HepG2 cells were transfected with siRNA at concentrations of 10 nM, using 0.1% (v/v) Lipofectamine RNAiMAX (Invitrogen, Paisley, UK), as described in the manufacturer’s protocol. Transfection reactions were performed in serum-free OptiMEM (Invitrogen, Paisley, UK). Cells were harvested after 48 h and used for total RNA isolation.

### RNA isolation and mRNA level determination

Total cellular RNA was isolated from the frozen liver samples and HepG2 cell pellets with a commercial RNA isolation kit (Total RNA Mini, A&A biotechnology, Poland). RNA concentration was determined on the basis of absorbance at 260 nm; all the samples showed 260/280 nm absorbance ratio of about 2.0. Prior to the reverse transcription, the RNA samples were treated with RNase-free DNase I (Fermentas, International Inc., Canada). First strand cDNA synthesis and the determination of mRNA levels by RT-PCR were performed as described previously [41], using a CFX Real-Time Detection System (Bio-Rad Laboratories Inc., USA). The primer sequences used in this study are presented in Table 1. β-actin mRNA was used as an internal standard. Relative quantities of the transcripts were calculated using the 2^−ΔΔCT^ formula [42]. The amplification of specific transcripts was further confirmed on the basis of the melting curve profiles.

**Table 1:** The sequences of primers used in this study

Gene	Primer sequence (5′–3′)
*HNF1α*	F: AAGATGACACGGATGACGATGG
	R: GGTTGAGACCCGTAGTGTCC
*HNF4α*	F: AAATGTGCAGGTGTTGACCA
	R: CACGCTCCTCCTGAAGAATC
*PCSK9*	F: TGGCTGCATGACATTGCTTCTC
	R: GCACTGGAGAACCACACAGG
*β-actin*	F: GAAATCGTGCGTGACATTAAG
	R: GCTAGAAGCATTTGCGGTGGA

### SDS-PAGE and immunoblotting

Frozen rat liver was homogenized in 20 mM Tris–HCl buffer (pH 7.8) containing 0.2% Triton X-100 and protease inhibitor cocktail (Sigma, USA), and then centrifuged (15 000 × *g*, 20 min, 20 °C). Aliquots of the obtained supernatants containing 10 µg of protein were separated by 10% SDS-PAGE and electroblotted to Immuno-Blot™ PVDF Membrane (Bio-Rad Laboratories, Hercules CA, USA). The membrane was blocked by incubation with blocking buffer, and then incubated with rabbit polyclonal anti- HNF4α antibody (NBP1-00876, Novusbio), mouse monoclonal anti-HNF1α antibody (GTX12064, GeneTex), rabbit polyclonal anti- LDL-Receptor antibody (AB30532, ABCAM), goat polyclonal anti-PCSK9 (AF3985-SP, R&D Systems), and rabbit polyclonal anti-actin antibody (A 5060, Sigma–Aldrich). Secondary HRP-conjugated antibodies were obtained from Sigma Aldrich (A0545, A9044, A5420). The reactions were visualized with a SuperSignal West Pico chemiluminescent substrate (Thermo Fisher Scientific Inc., Rockford, IL, USA). The bands (visible on the film after the chemiluminescent detection) were compared to molecular mass protein markers (SM1811) obtained from Fermentas, visible on the membrane after electroblotting. The film was adjusted to the membrane in such way that the membrane edges were visible on the film. Blots were analyzed using Quantity One program, version 4,0 (Bio-Rad).

### Statistical analysis

Statistical analysis was conducted using a MS Excel 2010 spreadsheet (Microsoft). All the data were expressed as mean values (± SD) for the controls and lipectomized rats. The significance of differences was analyzed with Student *t*-test. The differences were considered significant at *p* value < 0.05.

## Results

The effects of lipectomy on the mass of inguinal, retroperitoneal and epididymal WAT were previously reported [41]. Briefly, lipectomy resulted in a complete reduction of inguinal adipose tissue and approximately 80% reduction of retroperitoneal and epididymal adipose tissue as compared to the control rats. Consequently, the overall reduction of sum inguinal, retroperitoneal, and epididymal adipose tissue mass in the lipectomized rats corresponded to approximately 90%. However, one has to keep in mind that other anatomical WAT sites like mesenteric, gluteal, perirenal, and interscapular, whose corresponds to approximately 75–80% of total adipose tissue in rats [43], has not been dissected (except of mesenteric WAT). Mean baseline body weights of the controls and lipectomized rats were essentially similar (312 ± 18 g vs. 315 ± 19 g). Mean final body weights determined at the end of the experiment were 403 ± 21 g and 407 ± 17 g for the controls and lipectomized rats, respectively. Similar final body weight for the controls and lipectomized rats, suggested that partial lipectomy induces a compensatory increase of adipose tissue mass located in different anatomical sites, including visceral fat. Previously, we have observed significant increase of mesenteric WAT mass after lipectomy as compared to control rats [44]. It should be noted that in normal weight women abdominal liposuction also induces increase of visceral fat [45].

**Fig. 1:**
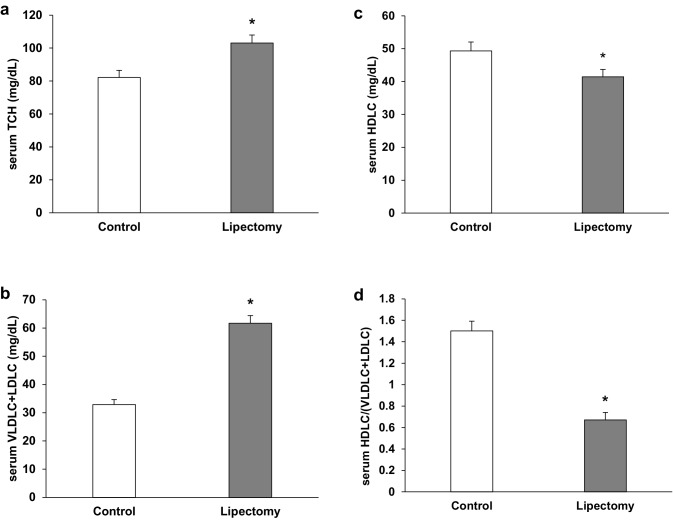
Serum cholesterol concentrations: **a** total serum cholesterol (TCH); **b** VLDL + LDL-cholesterol; **c** HDL-cholesterol and **d** serum HDLC/(VLDLC + LDLC) ratio of the controls and lipectomized rats. *a.u*. arbitrary units. Data are presented as mean ± SD. *p < 0.05

**Fig. 2:**
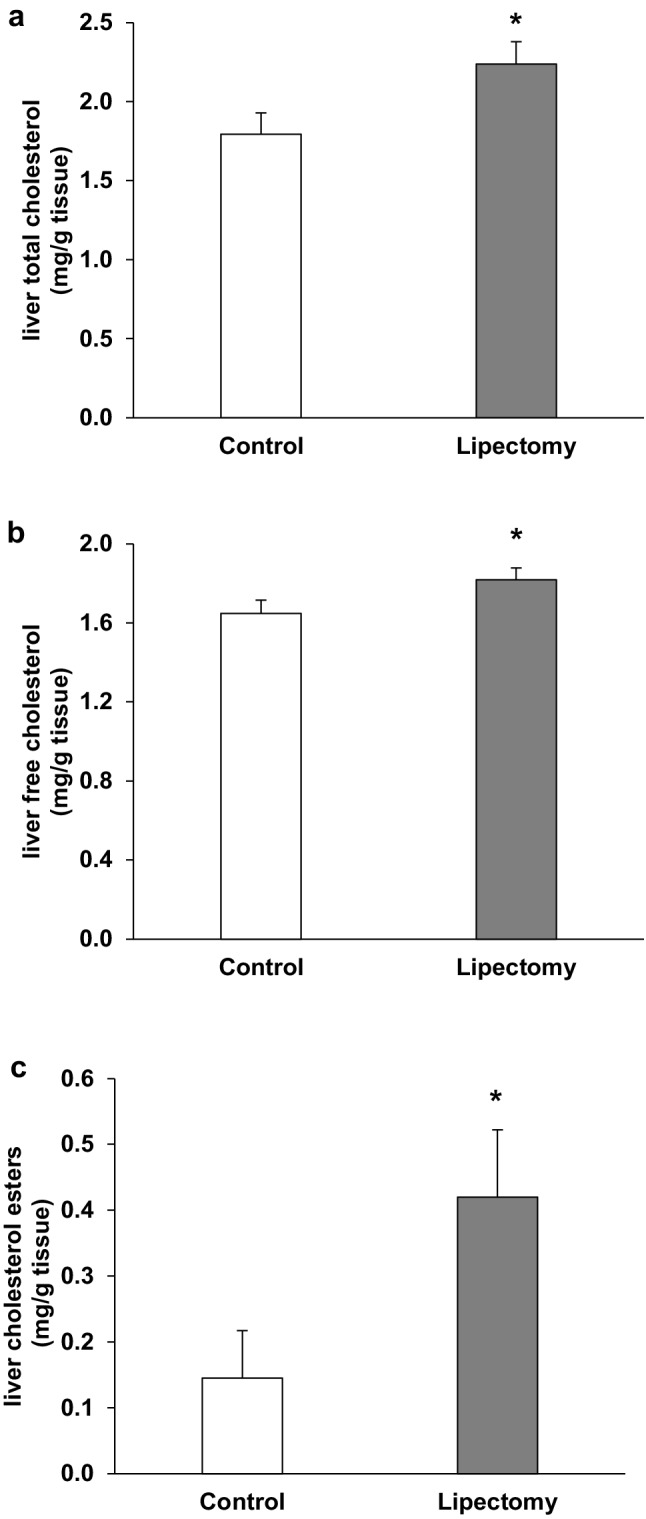
Liver cholesterol concentrations: **a** total liver cholesterol; **b** free cholesterol; **c** cholesterol esters of the controls and lipectomized rats. *a.u*. arbitrary units. Data are presented as mean ± SD. *p < 0.05

Partial lipectomy was reflected by an increase in the serum concentration of total cholesterol (Fig. 1a), and VLDL-cholesterol + LDL- cholesterol (Fig. 1b). Simultaneously, slight but statistically significant decrease in serum HDL-cholesterol concentration in lipectomized rats was found (Fig. 1c). Subsequently serum HDL-cholesterol/VLDL-Cholesterol + LDL—Cholesterol ratio significantly decreased in lipectomized rats as compared to control animals (Fig. 1d). The increase of serum total cholesterol and VLDL-cholesterol + LDL- cholesterol concentrations was associated with an increase in the liver tissue of: (a) total cholesterol (Fig. 2a, b) free cholesterol (Fig. 2b, c) cholesterol esters (Fig. 2c) content. Moreover there was a strong negative correlation between the increase of total serum cholesterol and weight of total fat that resembled after two surgeries (r = − 0.79; *p* < 0.05).

**Fig. 3:**
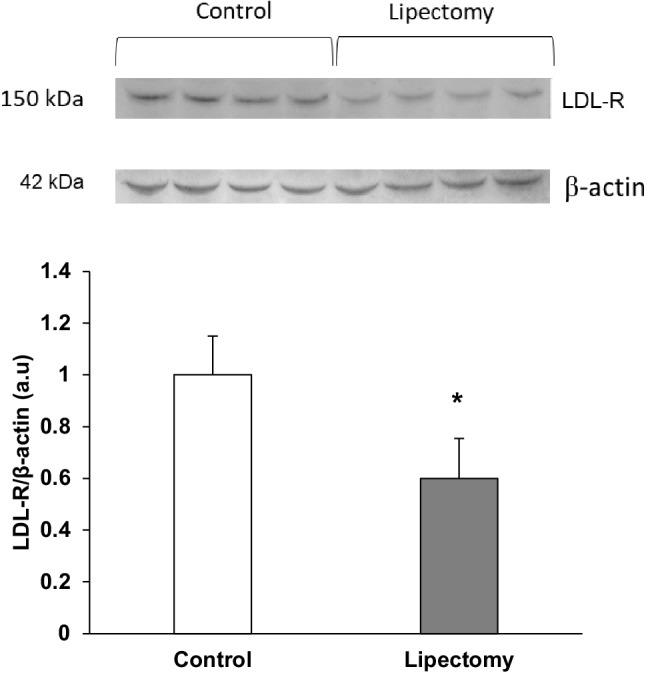
Western blot analysis of liver LDL-R: upper panel—representative western blot analysis of the controls and lipectomized rats; lower panel—densitometric analysis of western blot bands standardized against actin. *a.u*. arbitrary units. Data are presented as mean ± SD. *p < 0.05

**Fig. 4:**
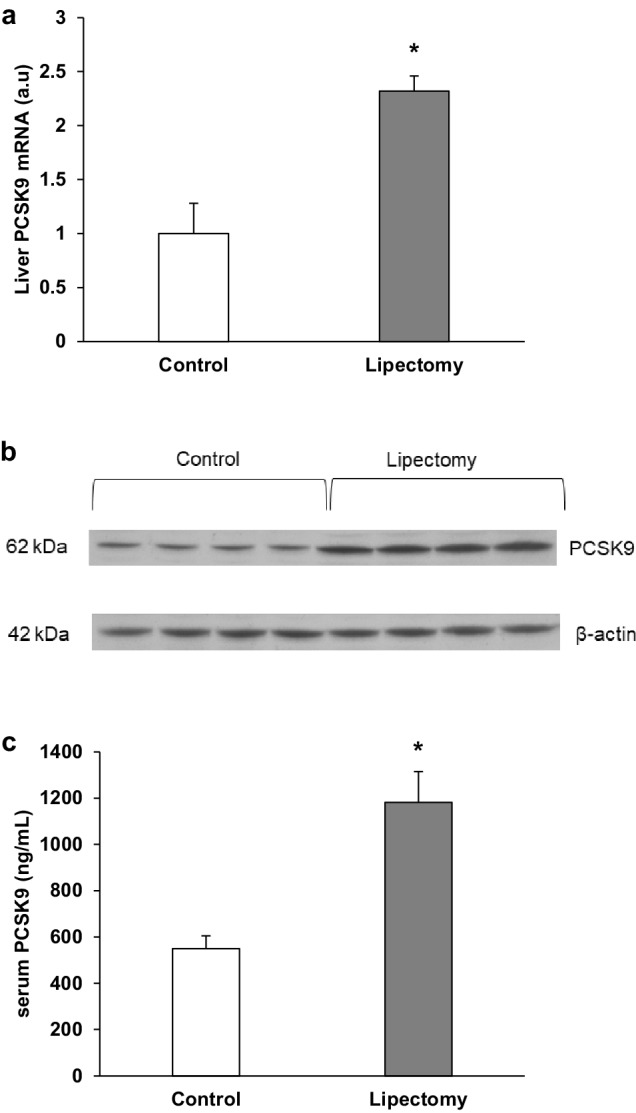
Analysis of PCSK9: **a** liver mRNA relative levels; **b** representative liver western blot protein analysis standardized against actin and **c** serum PCSK9 concentrations of controls and lipectomized rats. *a.u*. arbitrary units. Data are presented as mean ± SD. *p < 0.05

Changes in serum and liver cholesterol concentration after partial lipectomy were associated with significant decrease in the liver LDL-receptor (LDL-R) determined by Western blot (Fig. 3). The decrease in liver LDL-R was associated with higher level of PCSK9 mRNA in liver of lipectomized rats (Fig. 4a). Different liver expression levels of PCSK9 mRNA observed in the controls and lipectomized rats were reflected by intergroup differences in the liver levels of PCSK9 protein documented on Western Blot analysis (Fig. 4b) and serum PCSK9 concentrations (Fig. 4c). Thus, it is very likely that enhanced expression of gene encoding liver PCSK9 leads to higher serum PCSK9 concentration (Fig. 4d) and contributes to the decrease in the liver LDL-R (Fig. 3). In turn, these changes probably contribute to the increase in serum total and VLDL + LDL-cholesterol concentrations (Fig. 2). This assumption was supported by: (a) very strong negative correlation found between the serum PCSK9 concentration and the liver LDL-R level (*r* = − 0.92; *p* < 0.05) and (b) very strong positive correlation between serum VLDL-cholesterol + LDL-cholesterol and serum PCSK9 level (*r* = 0.95; *p* < 0.05).

**Fig. 5:**
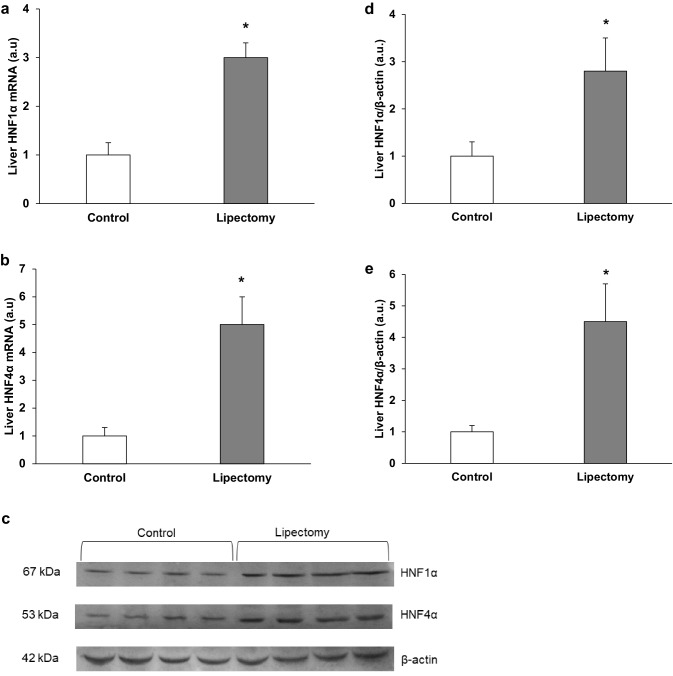
Analysis of HNF transcription factors in liver: **a** HNF1α mRNA relative levels; **b** HNF4α mRNA relative levels **c** representative western blot protein analysis of HNF1α and HNF4α standardized against actin of controls and lipectomized rats, **d** densitometric analysis of western blot HNF1α bands standardized against actin, **e** densitometric analysis of western blot HNF4α bands standardized against actin. *a.u*. arbitrary units. Data are presented as mean ± SD. *p < 0.05

As expected, partial lipectomy was reflected by approximately three-fold increase in the liver HNF1α (Fig. 5a) and about five-fold increase in HNF4α (Fig. 5b) mRNA levels. The different liver levels of HNF1α and HNF4α mRNA of the controls and lipectomized rats were reflected by intergroup differences in the HNF1α and HNF4α protein levels documented on Western Blot analysis (Fig. 5c—representative western blots and Fig. 5d, e—densitometric analysis of Western Blots bands).

**Fig. 6:**
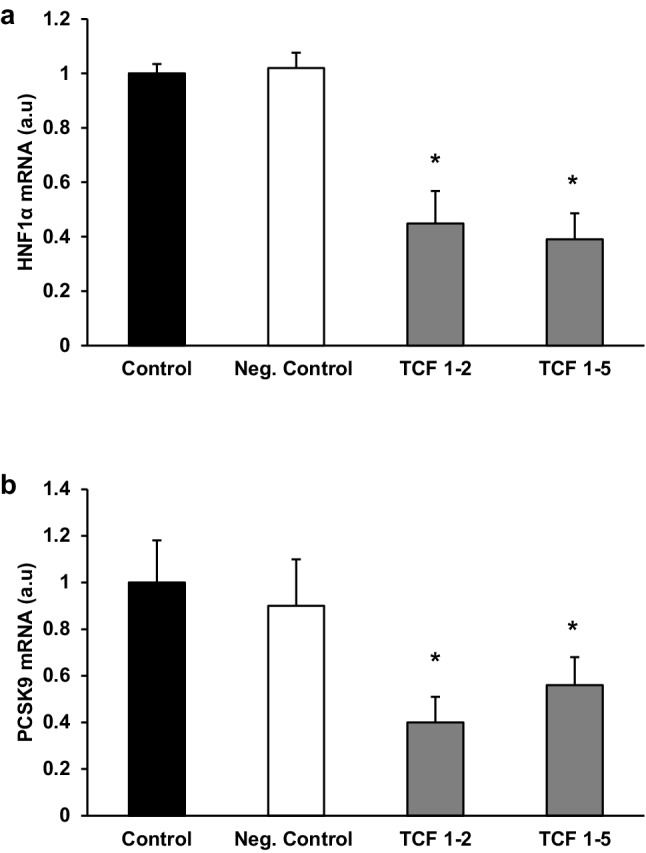
Subsequent inhibition of HNF1α and PCSK9 expression in HepG2 cells. silencing of HNF1α expression by two different siRNA (**a**) resulted in decrease of PSCK9 mRNA expression (**b**). Lipofectamine-treated cells were used as control. Graphs represent the mean ± SD of data from 6 cell plates in three separate experiments. *a.u*. arbitrary units.*p < 0.05

The pattern of changes in the liver HNFs mRNA and protein levels of the controls and lipectomized rats resembled the one observed in PCSK9 mRNA and protein levels (Figs. 4, 5, respectively). Moreover, strong positive correlations were found between the liver levels of PCSK9 and HNF1α mRNA (*r* = 0.82, *p* < 0.05). Thus one can suppose that up-regulation of HNF1α and HNF4α plays a key role in the increase of PCSK9 gene expression in lipectomized rats. To confirm this assumption HNF1α deregulation was performed and its effect on PCSK9 gene expression in hepatocyte cells (HepG2) was examined. As shown in Fig. 6 the decrease in HNF1α mRNA levels by two different siRNA (TCF 1–2 and TCF 1–5) was associated with the decrease in PCSK9 mRNA level. Together, the results presented above, especially parallel expression of PCSK9 and HNF1α both in vivo and in vitro, suggest that up-regulation of HNF1α contributes to the increase in liver PCSK9 gene expression and leads to hypercholesterolemia in lipectomized rats.

## Discussion

This study was the first to show that reduction of fat mass induced by the surgical removal of total inguinal and majority of retroperitoneal and epididymal adipose tissue in rats may be associated with up-regulation of gene encoding liver PCSK9 and subsequently with higher level of PCSK9 in serum. This in turn may lead to decrease in liver LDL-R and elevated serum cholesterol concentration. PCSK9 is a serine protease synthesized and released mainly by liver, which binds to LDL-R on the surface of hepatocyte, forming a complex that is internalized and degraded [46]. By promoting degradation of LDL-R, PCSK9 prevents its recycling to the hepatocyte membrane, leading to a substantial reduction of LDL-R level [47]. As shown in this paper, the increase in expression of gene encoding PCSK9 and increase in serum PCSK9 concentration (Fig. 4) were associated with the decrease in LDL–R level (Fig. 3) and increase in the serum and liver cholesterol concentrations (Figs. 1, 2). This suggests that the up-regulation of gene encoding PCSK9 in liver and subsequently elevated serum PCSK9 concentration may contribute to the decrease in liver LDL-R after lipectomy. Therefore, the changes in serum PCSK9 concentration and the decrease in liver LDL-R may contribute to the increase in the serum concentration of cholesterol after lipectomy. PCSK9 is expressed in other organs including lungs [48]. Thus, it is not excluded that production of PCSK9 by lungs contributes also to the increase in serum concentration of this protein. However, the effect of lipectomy on regulation of lung PCSK9 expression requires further studies.

Taking into account the results of previous studies in which HNF1α and HNF4α were shown to be transcriptional activators of gene encoding PCSK9 in liver [23, 25, 49], and our hereby presented findings, we hypothesize that these hepatocyte nuclear factors through transcriptional up-regulation of gene encoding PCSK9 might contribute to the hypercholesterolemia observed in lipectomized rats. The hypothesis is based on: (a) the fact that gene encoding PCSK9 is a target of HNFs [23, 25, 49], (b) strong positive correlation between *hnf1α* and *pcsk9* expression found in liver, (c) strong negative correlation between HNFs and LDL-R protein levels determined by western blot, and (d) strong negative correlation between serum PCSK9 concentration and liver LDL-R level.

Pivotal role of HNFs (especially HNF4α) in maintaining cholesterol homeostasis comes also from the experimental data obtained with liver *Hnf4α*^−/−^ mice. For instance, serum cholesterol concentrations were shown to be significantly reduced in liver specific *Hnf4α*^−/−^ mice [20]. Similarly, acute loss of liver *Hnf4α* in mice, generated by adenovirus expressing small hairpin RNA corresponding to *Hnf4α*, leads to hypocholesterolemia [21]. These results suggest that liver *Hnf4α* plays an important role in regulation of serum cholesterol concentrations. Yin et al. [21] suggests that the decrease in serum cholesterol concentration in mice after acute loss of liver *Hnf4α* is caused by inhibition of: (a) de novo cholesterol biosynthesis, (b) VLDL secretion, and (c) HDL formation. The results presented here indicate that HNFs may affect serum cholesterol concentration via regulation of serum PCSK9 level and consequently LDL-R level in hepatocytes. It means that up-regulation of liver HNFs in lipectomized rats may lead to increase of serum cholesterol concentration through decrease in LDL-R and increase in cholesterol synthesis.

In addition to its role in degradation of LDL-R, PCSK9 promotes degradation of VLDL-R [50] and fatty acid translocase (also known as CD36), what contributes to decrease in VLDL and long chain fatty acid [51] uptake. Thus, high level of serum PCSK9, promoting VLDL-R and CD36 degradation, could also contribute to elevated serum VLDL–cholesterol and FFA level (Fig. 1b) leading to hypertriglyceridemia found in lipectomized rats [17, 18].

As already mentioned in the “Introduction” section, up-regulation of gene encoding HNF4α was also observed in lipectomized obese rats [17]. Given the pivotal role of HNFs in up-regulation of gene encoding PCSK9, one would expect elevated concentration of serum PCSK9 and cholesterol. Surprisingly, lipectomy performed in this experimental model has no significant effect on serum concentration of total, LDL- and HDL-cholesterol [17]. Since serum PCSK9 concentrations (as well as *pcsk*9 regulation) has not been presented by Ling and co-workers [17], the reasons for the discrepancy between results presented here and Ling and co-workers [17] is unknown. We can speculate that different diets used in our experiments (normal laboratory diet) and in Ling and co-workers experiments (high fat and cholesterol diet) could be the reason.

Besides parallel up-regulation of genes encoding liver HNFs and PCSK9, the role of HNFs in regulation of gene encoding PCSK9 was confirmed by the results presented in Fig. 6, which shows that silencing of HNF1*α* with small interfering RNA (siRNA) led to the decrease in in PCSK9 mRNA levels in HepG2 cells. However, the reasons behind the increased expressions of *Hnf1α* and *Hnf4α* in the rat liver after surgical removal of adipose tissue are still unclear. The expression of gene encoding Hnf4α was postulated to be down-regulated by cytokines including IL-1β [52, 53], and removal of adipose tissue was shown to be reflected by a decrease in the circulating levels of pro-inflammatory cytokines [54, 55]. Thus, it can be hypothesized that surgical removal of adipose tissue in rats may result in a decrease in cytokine levels, which in turn leads to increase in the expression of *Hnf4α* gene.

In view of the pivotal role of HNF1α and HNF4α in the lipid metabolism and maintenance of serum cholesterol concentration in lipectomized rats, one may ask how these findings translate onto humans subjected to liposuction (or humans with lipodystrophy). At present we can only hypothesize that similar to the lipectomized rats, patients after liposuction may present with: (a) increased liver levels of HNF1α, HNF4α, and PCSK9; (b) increased circulating PCSK9 concentration; and (c) decreased liver level of LDL-R and resultant increase of serum cholesterol concentration. In fact, Ybarra et al. [12] showed that liposuction removal of subcutaneous abdominal fat in normal-overweight subjects results in an increase in circulating (a) total cholesterol; (b) LDL-cholesterol; and (c) ApoB-100 and LDL-cholesterol/ ApoB-100 ratio. This observation is in line with our hereby presented findings. Moreover, elevated concentrations of serum total and LDL-cholesterol concentrations were also found in some patients with lipodystrophy [13, 14]. However, in the most studies reported so far, abdominal liposuction did not have significant effect on circulating cholesterol concentration [3–7] or even decrease in the serum cholesterol concentrations of was found [8–11]. Moreover, no changes in serum total and LDL-cholesterol concentrations in some patients with lipodystrophy were also observed [15, 56]. Thus, at present it would be inappropriate to speculate if the changes taking place in humans subjected to liposuction are similar to those observed in our partially lipectomized rats.

**Fig. 7:**
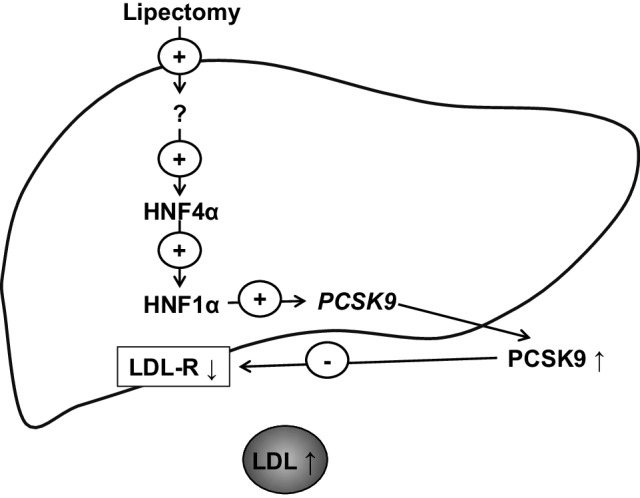
Proposed mechanism showing how HNFs may affect circulating LDL-cholesterol concentration in rats after lipectomy. Lipectomy causes increase of liver HNF4α and HNF1α. HNF1α activates *Pcsk9* gene promoter leading to increased concentration of plasma PCSK9. PCSK9 binds to LDL-R and impairs proper scavenging of serum LDL-cholesterol. For details see “Discussion”

In conclusion, our study showed that partial surgical removal of WAT in rats is associated with the coordinated up-regulation of liver genes encoding Hnf1α, Hnf4α and PCSK9. These changes are associated with increased circulating PCSK9 concentration, and decrease in liver LDL-R. Consequently, the post-lipectomy increase in circulating cholesterol concentration may result from decreased uptake of cholesterol by LDL-R in rat liver. The above mentioned mechanism of post-lipectomy hypercholesterolemia is summarized on Fig. 7. Although our findings provide a new insight into post-lipectomy catabolism of cholesterol in experimental model, further studies are needed to determine an association between hypercholesterolemia, circulating PCSK9 concentrations and up-regulation of genes encoding PCSK9, HNF1α, and HNF4α in humans subjected to liposuction or patients with lipodystrophy.
